# Histology of Pompia Peel and Bioactivity of Its Essential Oil: A New Citrus-Based Approach to Skin Regeneration

**DOI:** 10.3390/ph18091256

**Published:** 2025-08-24

**Authors:** Emma Cocco, Giulia Giorgi, Valeria Marsigliesi, Francesco Mura, Jorge M. Alves-Silva, Mónica Zuzarte, Lígia Salgueiro, Valentina Ghiani, Enrico Sanjust, Danilo Falconieri, Delia Maccioni, Alessio Valletta, Elisa Brasili, Andrea Maxia

**Affiliations:** 1 Department of Environmental Biology, Sapienza University of Rome, P.le Aldo Moro 5, 00185 Rome, Italy; giulia.giorgi@uniroma1.it (G.G.); f.mura@uniroma1.it (F.M.); alessio.valletta@uniroma1.it (A.V.); elisa.brasili@uniroma1.it (E.B.); 2 Faculty of Medicine, Coimbra Institute for Clinical and Biomedical Research (iCBR), University of Coimbra, Azinhaga de S. Comba, 3000-548 Coimbra, Portugal; jmasilva@fmed.uc.pt (J.M.A.-S.); mzuzarte@uc.pt (M.Z.); 3 Faculty of Pharmacy, University of Coimbra, Azinhaga de S. Comba, 3000-548 Coimbra, Portugal; 4 Department of Chemical Engineering, Chemical Engineering and Renewable Resources for Sustainability (CERES), University of Coimbra, 3030-790 Coimbra, Portugal; 5 Via Piccioni 38, 09124 Cagliari, Italy; 6 Department of Biomedical Sciences, University of Cagliari, 09042 Monserrato, Italy; 7 Laboratory of Economic and Pharmaceutical Botany, Department of Life and Environmental Sciences, University of Cagliari, V.le S. Ignazio da Laconi 13, 09123 Cagliari, Italya.maxia@unica.it (A.M.)

**Keywords:** *Citrus* genus, Pompia, microscopy, histological characterization, GC-MS, essential oil, wound healing, senescence, skin regeneration

## Abstract

**Background/Objectives**: Pompia is an ancient, endemic citrus ecotype native to Sardinia (Italy), characterized by distinctive morphology and high content of bioactive compounds. Despite increasing interest, several aspects of this fruit, including its histological characteristics, remain poorly understood. This study aims to address this gap by investigating the anatomical features and spatial distribution of secretory cavities involved in essential oil (EO) production and accumulation, while also evaluating the EO’s chemical profile and associated biological activity. **Methods**: Pompia peel (flavedo and albedo) was subjected to histological analysis through fixation, dehydration, resin inclusion and sectioning. Sections were stained with 0.05% toluidine blue and observed under a light microscope to measure different parameters of secretory cavities. Essential oil (EO) was obtained from Pompia peel by hydrodistillation and characterized by gas chromatography–mass spectrometry (GC–MS) analysis. The biological activity of Pompia EO was assessed in vitro using NIH/3T3 fibroblasts, where wound-healing was evaluated by scratch assay and anti-senescence effects by β-galactosidase and γH2AX activity. **Results**: Microscopic analysis of the peel revealed pronounced variability in depth and size of the secretory cavities, along with the presence of lenticel-like structures in the epidermis. GC–MS analysis showed that Pompia EO is dominated by limonene (89%), with minor compounds including myrcene, geranial and neral. In vitro biological assays demonstrated that the EO promotes cell migration in a wound-healing model at concentrations ≥ 12.5 µg/mL and reduces markers of cellular senescence, including β-galactosidase activity and γH2AX foci, in etoposide-induced senescent fibroblasts. **Conclusions**: Overall, this study provides the first histological characterization of Pompia peel and confirms the bioactive potential of its EO. These findings support future applications in skin regeneration and anti-aging strategies and contribute to the valorization of this underexplored *Citrus* ecotype.

## 1. Introduction

“Pompia” is the popular name of an ancient *Citrus* ecotype of probable hybrid origin, belonging to the Rutaceae family. It is endemic to the historical region of Baronia in Central-Eastern Sardinia, where it has been traditionally cultivated.

It was first documented in Sardinia at the end of the 18th century, both in agronomic essays and official historical documents [1,2]. In 1837, Moris [3] described its distinctively irregular and rough-textured fruit and assigned the botanical name *Citrus medica* L. var. *monstruosa* Moris. More recently, different taxonomic designations have been proposed, including *C. limon* (L.) Osbeck var. *pompia* Camarda var. nova [4] and *C. medica* L. var. *tuberosa* Risso and Poiteau [5], reflecting the ongoing interest in its classification and botanical identity.

The fruit represents the most distinctive morphological trait of the plant: a medium-large, deep yellow hesperidium characterized by a deformed knobbly peel and a thick mesocarp [4]. It is characterized by a low pulp content and an acidic taste, making it unsuitable for raw consumption. Nevertheless, it holds a significant role in the Sardinian local economy and popular traditions. In fact, Sardinia accounts for 2% of Italy’s total *Citrus* production. However, this percentage rises to 22% when considering hybrid varieties, among which Pompia is the most significant [6].

Within local traditions, Pompia is commonly used for producing traditional liqueurs and sweets consumed during celebrations. Nothing is wasted: the albedo is cooked with honey in “Pompia intrea,” while the flavedo is caramelized in “S’aranzada.” Its bitter pulp is also used in juices, condiments and marmalades [7]. Recognized as a Traditional Food Product of Sardinia (P.A.T.) and a Slow Food Presidium since 2004 [8], Pompia’s cultural and biodiversity value is protected, promoting sustainable practices across its supply chain.

Furthermore, Pompia fruit is characterized by the presence of various bioactive compounds and offers multiple potential applications [9]. The essential oil, obtained through the distillation of the outer peel (flavedo), is rich in monoterpenes. Among these, limonene is the most abundant, accounting for approximately 75% to 90% of the total composition. Other compounds, present in smaller quantities, include myrcene, geranial, neral and α-pinene [10,11]. Hydroalcoholic extracts obtained from the outer peel are particularly rich in phenolic acids and flavonoids. Among these, quinic acid and sinapic acid are the most abundant phenolic acids, while the predominant flavonoids include naringin, neoeriocitrin and neohesperidin [12]. Both Pompia’s essential oil and peel extracts demonstrate various antioxidant and antimicrobial activities and represent the most thoroughly investigated component of the fruit [13,14,15,16].

Furthermore, analysis of the traditional dessert “Sa Pompia Intrea” revealed that its bioactive compounds remain stable even after prolonged cooking (5 to 6 h) [17]. This traditional sweet maintains a high limonene content in the volatile fraction (more than 70%). In contrast, in the polar fraction, several derivatives of cinnamic acid, limocitrol and limocitrin were identified, along with a variety of glycosylated flavonoid compounds [17]. These compounds have exhibited intrinsic antioxidant activity, and, consistently, in vitro cellular assays on the dessert have consistently demonstrated a notable free radical scavenging capacity and effective inhibition of cholesterol and fatty acid oxidation [17].

Notably, the essential oil has been tested for numerous bioactivities in the oral health and food fields. EO extracted from the leaves demonstrated bactericidal and antifungal effects against *Listeria monocytogenes* [18] and *Penicillium digitatum* [19]. Additionally, it has also been shown to inhibit the proliferation of HeLa and B16F10 cancer cells [14] and to interfere with the development of polymicrobial biofilms formed by *Pseudomonas aeruginosa* and pathogenic fungi [20]. This property is highly relevant for preventing respiratory, mucosal and oral infections. In the context of oral health, the EO has also been tested within lipid vesicles for the prevention of skin and oral infections, showing significant activity against *Escherichia coli*, *Pseudomonas aeruginosa*, *Staphylococcus aureus* and *Candida albicans* [16]. However, these results contrast with those of Flamini et al. (2019), who observed no significant antibacterial or antifungal activity against environmental bacteria, dermatophytes and fungi [11].

Given the contradictory results among different studies and the recognized potential of Pompia essential oil, along with its widespread local availability, further investigation is necessary to determine its full range of possible applications.

Anatomical studies focusing on the external peel (flavedo) are common within the *Citrus* genus, characterized by specialized secretory cavities responsible for the synthesis and accumulation of essential oils and other secondary metabolites. These specialized structures play a crucial role in defining the fruit’s aromatic profile and commercial value. Previous research demonstrated that morphometric parameters of secretory cavities can be used to predict optimal harvest timing and are often correlated with higher EO content, thereby enhancing yield and production efficiency [21,22]. Thus, histological characterization not only contributes to a better understanding of the anatomical basis for bioactive compound production but also supports the development of agronomic strategies and industrial applications.

In this context, a comprehensive histological analysis of the Pompia peel has been undertaken for the first time. Accordingly, the present research aims to provide a thorough characterization of Pompia fruit through an integrated approach based on histological, chemical and biological analyses. A detailed histological investigation of the peel provides critical insights into the development and functional anatomy of secretory structures, with potential applications in both agronomic practices and industrial processing. The obtained EO was analyzed by gas chromatography–mass spectrometry (GC-MS) and subsequently subjected to biological activity assessment, namely wound healing and anti-senescence potential. The results highlight a notable bioactive potential of Pompia essential oil, particularly its regenerative and anti-aging properties that may support innovative therapeutic strategies for chronic wounds and age-related skin disorders. Together, these findings enhance our understanding of this endemic *Citrus* variety and its potential uses, laying the foundation for future valorization efforts in both scientific and industrial contexts.

## 2. Results

### 2.1. Morphological and Histological Profile of Pompia Fruit

The morphological and histological analysis focused on Pompia peel, composed of flavedo and albedo, characterized by its wrinkled appearance. The examined fruits weighed approximately 400 g each. The flavedo, consisting of the outer colored portion of *Citrus* fruits from which essential oils are typically extracted, accounted for 36% (*w*/*w*) of the fruit. The predominant part was the pulp, making up 44% (*w*/*w*) of the fruit, while the albedo represented the minor portion, accounting for 11% of the fruit (*w*/*w*).

**Figure 1:**
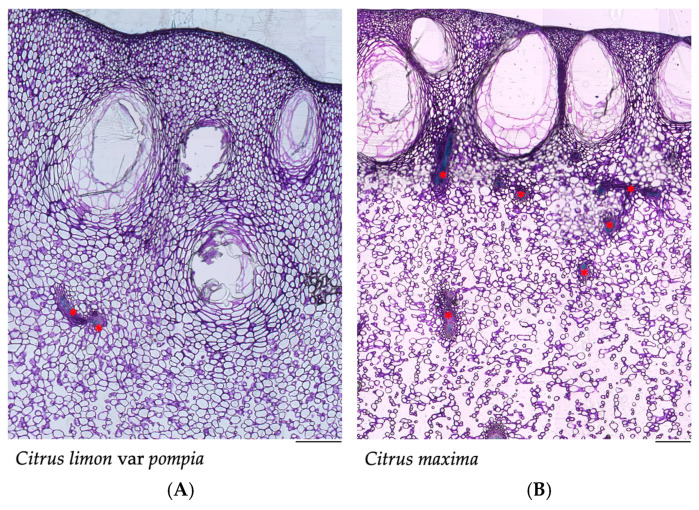
A representative section of the histological cross-section of (**A**) Pompia fruit and of (**B**) *Citrus maxima*. Red asterisks (*) indicate vascular bundles. Scale bar = 500 µm (×4).

After fixation and staining, the outer portion of the flavedo was observed under a light microscope. A representative section of the Pompia fruit (Figure 1A) and *C. maxima* (Figure 1B) is presented.

**Figure 2:**
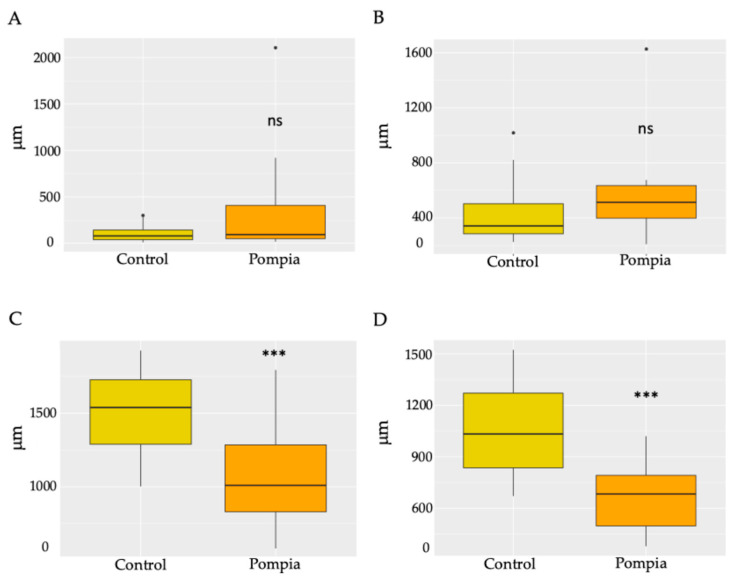
Box plot representing the measured histological parameters. Within each box, horizontal black lines denote median values; boxes extend from the 25th to the 75th percentile of each group’s distribution of values; vertical extending lines denote adjacent values (i.e., the most extreme values within 1.5 interquartile range of the 25th and 75th percentile of each group); dots denote observations outside the range of adjacent values: (**A**) distance of the secretory cavities from the epidermis, (**B**) distance of the secretory cavities from the vascular bundles, (**C**) height of the secretory cavities and (**D**) width of the secretory cavities. *Citrus maxima samples* are shown in yellow, and Pompia samples are shown in orange. *** *p* < 0.001; ns: not significant.

The fruits were subjected to histological analyses aimed at characterizing different aspects of the secretory cavities, including their shape, size, position and connection with the vascular bundles. Pompia showed a greater variability in relation to the distance of the secretory cavities from the epidermis, ranging from 16 μm to 2100 μm (Figure 2A). In contrast, *C. maxima* showed greater homogeneity, with superficially positioned cavities between 9 µm and 216 μm. In relation to size, Pompia secretory cavities were found to be smaller in both height and width (Figure 2C,D) compared to *C. maxima*. Finally, toluidine blue staining highlighted the vascular bundles, responsible for supplying water and nutrients to the fruit during ripening. Therefore, the distance from the vascular bundles was also considered, revealing, once again, more variability in Pompia (Figure 2B).

**Figure 3:**
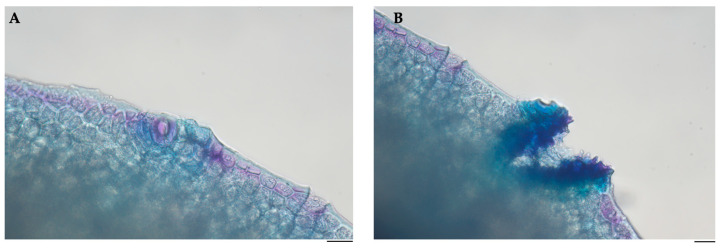
Representative histological cross-section of Pompia peel showing the outer epidermal layer. (**A**) In the central region, a stomatal opening is clearly visible, surrounded by epidermal cells. (**B**) Lenticel-like structure, characterized by a localized discontinuity in the epidermis. Scale bar = 50 µm (×40).

The histological analysis revealed distinctive features in the epidermis of Pompia. Specifically, localized thickening was observed at the level of the stomatal apertures (Figure 3A), and discontinuities were observed in the epidermal layer (Figure 3B). These discontinuities exhibit a morphology reminiscent of lenticels.

### 2.2. Essential Oil Analysis

The flavedo, the outer layer of the citrus peel, was utilized for the extraction of essential oil via three separate hydro-distillations. The following GC-MS analysis (Table 1) revealed that the main constituent of the essential oil was the monoterpene limonene (89%), followed by myrcene (1.71%), geranial (1.63%) and neral (1.24%). An example of the chromatographic run is provided in the Supplementary Materials (Figure S1).

### 2.3. Biological Activities

#### 2.3.1. Safety Profile of Pompia Essential Oil

**Figure 4:**
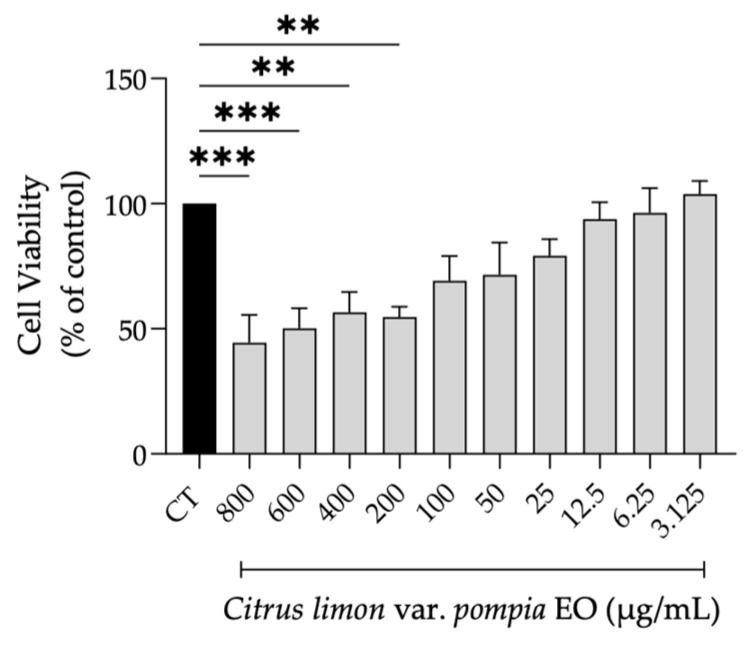
Effect of Pompia essential oil on the viability of NIH/3T3 fibroblasts. Bars represent the mean ± SEM of at least three independent experiments performed in duplicate. ** *p* < 0.01, *** *p* < 0.001, compared to control (untreated) cells.

Envisioning a putative biomedical use for Pompia essential oil, we first determined its effect on NIH/3T3 fibroblasts’ viability. As shown in Figure 4, concentrations below 100 µg/mL did not result in statistically significant differences in cell viability compared to control cells. Nevertheless, concentrations starting from 12.5 µg/mL were selected for biological assays, as they maintained cell viability above 80%.

#### 2.3.2. Effect of Pompia Essential Oil on Cell Migration

**Figure 5:**
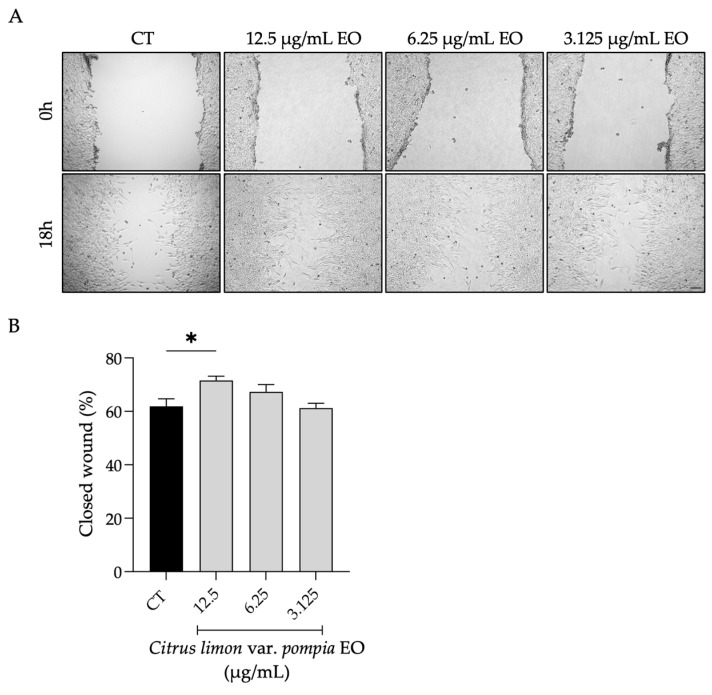
Effect of Pompia EO on NIH/3T3 fibroblasts’ cell migration. (**A**) Representative bright-field images of NIH/3T3 fibroblasts at 0 h and 18 h post-scratch; scale bar = 100 µm (×10). (**B**) Percentage of wound closure after 18 h of wound induction of NIH/3T3 fibroblasts in the presence (grey bars) and absence (black bar) of Pompia EO. Bars represent the mean ± SEM of at least three independent experiments performed in duplicate. * *p* < 0.05, compared to control (untreated) cells.

Following 18 h of wound induction, Pompia EO was able to increase cell migration in a dose-dependent manner, as shown in Figure 5. Statistical significance was observed at 12.5 µg/mL, where the percentage of wound closure was 71%, compared to 61% in control cells (*p* = 0.0371, R squared = 0.4376, 95% CI [−18.81, −0.5377]). Although the percentage of wound closure was 87% (data not shown) for allantoin, the concentration used for the essential oil was much lower (12.5 µg/mL vs 50 µg/mL), which highlights the strong biological effects of Pompia EO even at low concentrations.

#### 2.3.3. Effect of Pompia Essential Oil on Cellular Senescence

**Figure 6:**
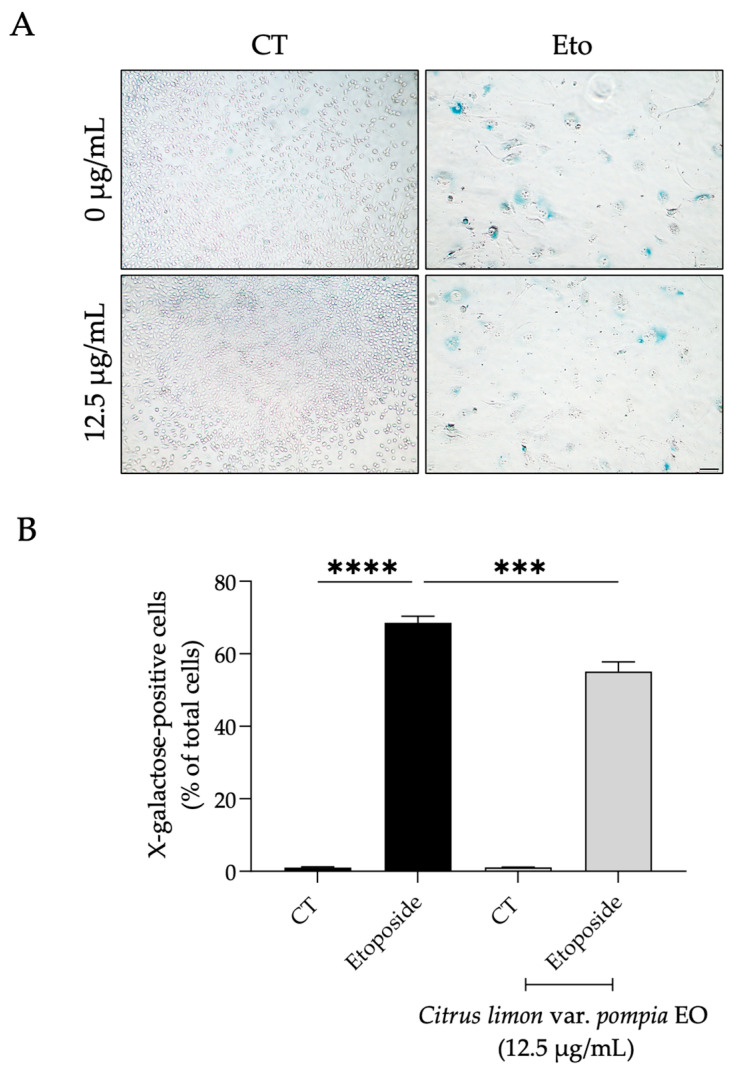
Effect of Pompia EO on etoposide-induced beta-galactosidase activity. (**A**) Representative bright-field images of NIH/3T3 fibroblasts, treated with etoposide (12.5 µM) for 24 h and left to recover for 7 days in the presence or absence of Pompia EO. The blue staining indicates positive cells for β-galactosidase; scale bar = 100 µm (×10). (**B**) Percentage of X-galactose-positive cells in NIH/3T3 fibroblasts treated with etoposide (12.5 µM) for 24 h and left to recover for 7 days in the presence (gray bars) or absence (black bars) of Pompia EO. Bars represent the mean ± SEM of at least three independent experiments, in which a minimum of 100 cells were counted. *** *p* < 0.001, **** *p* < 0.0001, compared to cells treated with etoposide alone (black bar).

As expected, fibroblasts treated with etoposide for 24 h and left to recover for 7 days in culture medium alone showed a significant increase in the percentage of cells positive for X-galactose (*p* < 0.0001, R squared = 0.9920, 95% CI [61.49, 73.56]) as observed by their distinct blue color (Figure 6A,B). Interestingly, when Pompia EO was added during the recovery phase, a significant decrease in the number of X-galactose-positive cells was observed (*p* = 0.0002, R squared = 0.9920, 95% CI [7.433, 19.50]) in comparison to cells treated only with etoposide (Figure 6A,B).

**Figure 7:**
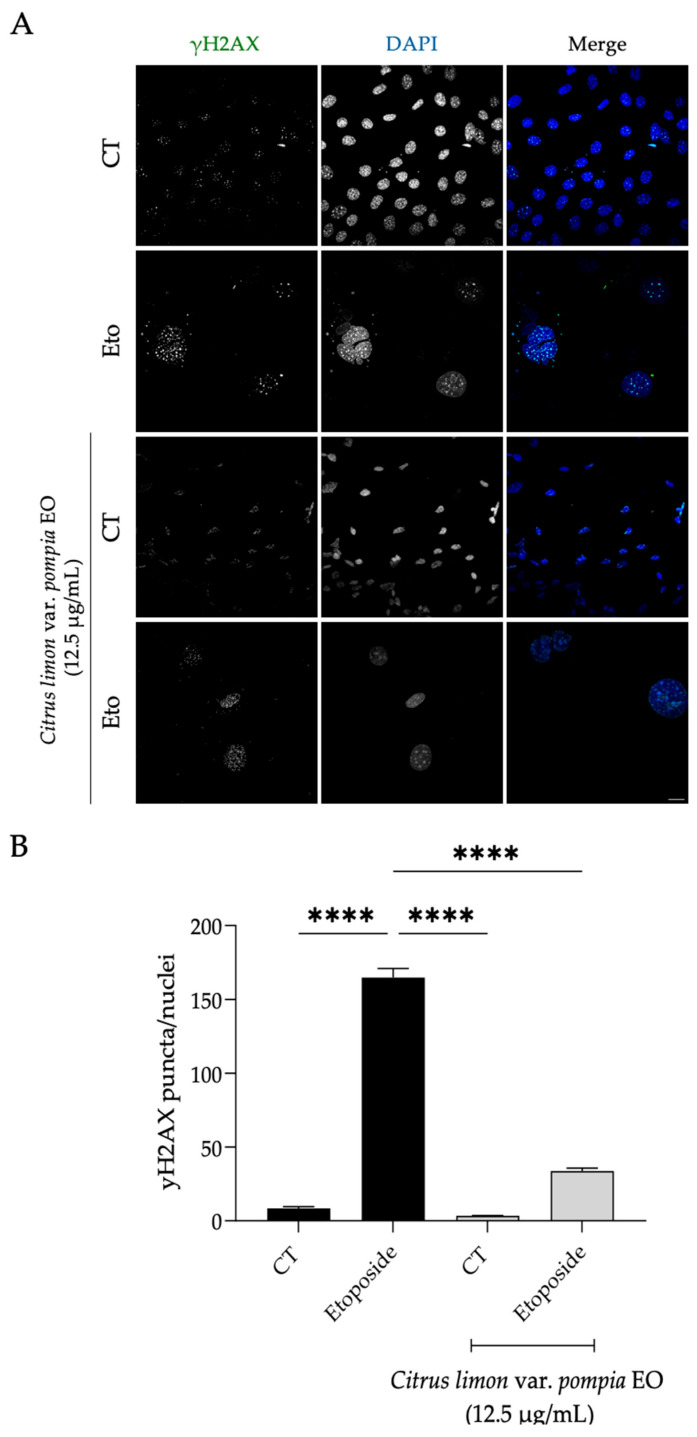
Effect of Pompia EO on etoposide-induced double-strand DNA breaks. (**A**) Representative confocal microscopy images of NIH/3T3 fibroblasts treated with etoposide (Eto; 12.5 µM) for 24 h and left to recover for 7 days in the presence or absence of Pompia EO. γH2AX was stained with Alexa Fluor 647, and nuclei were counterstained with DAPI; scale bar = 20 µm (×63). (**B**) Percentage of γH2AX puncta per nucleus in NIH/3T3 fibroblasts treated as described above. Gray bars represent cells treated with Pompia EO during the recovery phase; black bars indicate cells treated with etoposide only. Bars represent the mean ± SEM of at least three independent experiments, in which a minimum of 100 cells were counted. **** *p* < 0.0001 when compared to cells treated with etoposide only (black bar).

Accordingly, an increase in the number of γH2AX puncta in the nucleus of etoposide-treated cells was evident (*p* < 0.0001, R squared = 0.9949, 95% CI [142.7, 169.9]), as was as an increased nuclear area (Figure 7A), corroborating the previous results and confirming that the cells are indeed senescent. Strikingly, the presence of the essential oil greatly decreased the number of γH2AX puncta per nucleus (Figure 7A,B; *p* < 0.0001, R squared = 0.9949, 95% CI [117.5, 144.6]), thus suggesting the Pompia EO might facilitate the repair of double-strand damage induced by etoposide. Furthermore, the nuclei of Pompia EO-treated cells exhibited a more normal, control-like appearance and size compared to those treated with etoposide alone (Figure 7A), once again suggesting that the oil has anti-senescence potential.

## 3. Discussion

This study provides a comprehensive analysis of the Pompia fruit through a detailed characterization of the secretory cavities, followed by a chemical analysis of the essential oil and an assessment of its potential biological activities. The widespread local use of Pompia, deeply embedded in the Sardinian traditions and culture, along with its unknown origin and distinctive rough, irregular appearance, has contributed to growing interest in this fruit. To our knowledge, this is the first study to investigate the histological structure of the Pompia fruit, specifically analyzing the outermost portion of the flavedo and external part of the albedo. Previous scientific investigations primarily focused on the external morphology to characterize its features and determine its taxonomic affiliation [4,5].

Pompia peel, as common among *Citrus* fruits, is characterized by the presence of secretory cavities, where the essential oil is produced and accumulated. The histological analysis revealed that the secretory cavities in Pompia were smaller in both width and height compared to *Citrus maxima*. Additionally, in *C. maxima*, secretory cavities were consistently positioned adjacent to the epidermis, while Pompia showed marked variability in their spatial distribution relative to the epidermal layer. Such variability is atypical for *Citrus* species, where secretory cavities are generally located in close proximity to the epidermis. In fact, secretory cavities originate from an epidermal cell and an underlying one, finding themselves in a sub-epidermal position, linked with a stalk-like structure [25]. Knight et al. (2001) reported that oil glands and the epidermis are divided by a few cell layers [26]. However, they also highlighted how oil glands can change their orientation with respect to the epidermis during ripening. Considering the drastic change observed in the epidermis surface of Pompia during ripening, characterized by its high and deeper roughness, it is plausible to hypothesize a similar superficial sub-epidermal origin, along with the development of a pronounced tissue separation during fruit ripening.

The sub-epidermal position typically described is directly linked to the function of the secretory cavities, which produce secondary metabolites that characterize the fruit. Their proximity to the epidermis is crucial for optimal release to the external environment, protecting the plant against arthropod herbivores and contributing to its characteristic aroma [27].

Another crucial factor is fruit vascularization. During ripening, the fruit loses its photosynthetic capacity, which is associated with the color change from green to yellow/orange, transitioning from a source to a sink organ [28]. Therefore, the presence of vascular bundles plays a key role in the transport of photosynthates, water and ions. Previous studies have shown that enhanced vascularization at the pedicel, corresponding to its thickening, influences fruit quality [28]. In this study, we observed a slight increase in the distance between the secretory glands and the vascular bundles in Pompia fruit compared to *Citrus maxima*. This distance from the vascular bundles can be indicative of reduced vascularization of the secretory glands, which correspondingly exhibit a smaller size. In addition, *Citrus* fruit vascular bundles are present in the albedo section and are responsible for juice accumulation and, consequently, pulp development [29]. Therefore, the reduced vascularization in the inner albedo of Pompia should be further investigated to evaluate potential vascular abnormalities in this hybrid, which is characterized by a diminished, acidic and bitter internal pulp, making it unsuitable for raw consumption. Moreover, the differences herein reported should be confirmed with a more extensive comparison with other types of *Citrus* fruit.

Microscopic analysis also allows the observation of lenticel-like structures in the outer epidermal layer. Similar phenomena, such as fruit cracking, are well-documented in *Citrus* species [30,31,32]. Considering this observation, the superficial location could render them susceptible to mechanical rupture due to internal expansion forces associated with fruit growth and maturation, given Pompia’s characteristically rough and uneven surface. Kaur et al. (2019) linked this disorder to physiological imbalances affecting vascular function, including reduced water and mineral transport [28]. Accordingly, we hypothesize that these epidermal features are linked to the fruit’s vascularization. This hypothesis is supported by our observation of reduced vascular bundle density near the secretory cavities, indicating that limited vascular supply may contribute to both morphological irregularities and altered secretory tissue development in Pompia.

The GC-MS analysis confirms that Pompia EO is characterized by high proportions of limonene (89%), consistent with previous reports indicating values between 75% and 95% [4,10,11,14]. As demonstrated by Fenu et al. (2010) [10], such a variation range is closely related to the stage of fruit development, with maximal concentrations occurring at full ripeness and a progressive decline thereafter, likely reflecting shifts in the metabolic balance between limonene and its precursors. In line with this interpretation, the peak in limonene is accompanied by a marked reduction of biosynthetic intermediates such as geraniol and β-ocimene, which remain below 1% in the present study. More broadly, limonene is generally recognized as the predominant constituent of *Citrus* essential oils, although substantial interspecific variation exists. Comparative data indicate limonene levels of 50–60% in *Citrus reticulata* (mandarin) and 37–70% in *C. limon* (lemon) and that limonene reaches higher concentrations, ranging between 70 and 90%, in *C. aurantium* (sour orange) [33].

Considering previous studies attributing effective antimicrobial properties to Pompia essential oil against bacteria and fungi commonly involved in acute and chronic skin wounds, namely *Pseudomonas aeruginosa*, *Staphylococcus aureus* and *Candida albicans* [16], we also evaluated its impact on wound healing using a migration assay. Our results demonstrate that Pompia essential oil, at a relatively low concentration (12.5 µg/mL), significantly enhanced wound healing in skin fibroblasts. The observed 10% wound-healing rate is quite relevant in chronic wounds (e.g., diabetic foot), where even small improvements have clinical significance, and during aging, as older individuals naturally present slower healing rates. This finding is consistent with a previous study carried out by Palmas et al. (2020) [34], which reported that essential oils extracted from Pompia leaves and encapsulated in phospholipid vesicles accelerated the repair of keratinocyte wounds, although higher concentrations of the oil were used. Other species of *Citrus* have also demonstrated promising wound-healing properties. For instance, the essential oil extracted from *Citrus* × *limon* ‘Eureka’ was found to promote wound healing in keratinocytes at concentrations similar to those reported in the present study [35]. Moreover, in an in vivo wound-healing model, treatment with *Citrus reticulata* essential oil resulted in a significant reduction in wound diameter [36]. Interestingly, these essential oils, similar to those of Pompia, are rich in limonene, a compound that has been shown to exert several beneficial effects on the skin. Indeed, limonene improved tissue regeneration in a mechanical skin lesion model, probably by inducing neovascularization [37]. Also, in a diabetic model, limonene enhanced wound healing by promoting tissue re-epithelialization, and a reduced inflammatory response was observed in the wounds of treated mice [38]. In another skin wound in vivo model, limonene promoted tissue regeneration by increasing wound contraction and re-epithelization [39]. Moreover, chitosan-coated poly(lactic acid) nanofibers loaded with limonene increased fibroblasts’ adhesion and proliferation [40], further supporting the potential of this compound as a wound-healing agent and justifying the therapeutic relevance of oils rich in limonene. Furthermore, liposomes containing citral, a mixture of geranial and neral, both of which are also found in Pompia essential oil, enhanced wound healing in keratinocytes when compared to free citral [34]. Another study reported that nanofibers containing citral promoted fibroblast proliferation [41], suggesting its potential role in wound healing. Overall, the observed wound-healing properties of Pompia essential oil may result from a synergistic interaction between both major and minor compounds, such as limonene, myrcene, geranial and neral, each of which has been individually reported to promote wound-healing effects.

Cellular senescence plays a critical role in various stages of the wound-healing process, influencing both its progression and overall outcome. While transient senescence contributes positively to tissue repair by regulating inflammation and promoting regeneration, the prolonged or excessive accumulation of senescent cells can hinder wound closure and is associated with chronic, non-healing wounds [42]. Given the relation between wound healing and cellular senescence, we aimed to investigate the potential effect of Pompia essential oil on fibroblast senescence induced by etoposide, a topoisomerase II inhibitor, that induces stable double-strand breaks (DSBs), activating key kinases that lead to cell cycle arrest and eventual senescence [43,44]. We demonstrated that Pompia essential oil exhibits anti-senescence properties, as evidenced by a reduction in senescence-associated β-galactosidase (SA-β-gal) activity and a reduced number of γH2AX foci per nucleus. These findings suggest that the essential oil may facilitate the repair of DNA double-strand breaks. To the best of our knowledge, this is the first study to report the anti-senescence potential of Pompia essential oil, opening new avenues for its application in anti-aging strategies. Nevertheless, anti-aging effects have been reported for other *Citrus* species. For instance, essential oils derived from *Citrus paradisi*, *C. sinensis* and *C. deliciosa* inhibited the activity of key enzymes involved in skin aging, including tyrosinase, elastase and collagenase [45]. Similarly, *C. aurantium* essential oil demonstrated inhibitory effects on both elastase and collagenase [46]. Essential oil from *C. reticulata* also inhibited hyaluronidase, collagenase and amylase activity [47]. Moreover, the essential oil of *Citrus* × *limon* ‘Eureka’ was found to enhance several antioxidant markers in an animal model of aging [48]. Interestingly, limonene, the major constituent of these essential oils, has been identified as a potential anti-aging agent, as previously reviewed [49]. In addition, another study demonstrated that limonene exerts anti-aging effects by protecting keratinocytes from hydrogen peroxide-induced oxidative damage [50]. Besides limonene, other compounds present in Pompia essential oil have demonstrated beneficial skin effects. For example, myrcene has been shown to protect keratinocytes from hydrogen peroxide-induced oxidative stress [51], suggesting its potential as an anti-photoaging agent. Moreover, essential oils rich in citral have been reported to reduce senescence-associated β-galactosidase activity in a model of doxorubicin-induced cellular senescence [52]. Therefore, the anti-aging effects observed for Pompia essential oil may, at least in part, be attributed to the synergistic activity of bioactive constituents, including limonene, myrcene, geranial and neral.

Overall, the promising healing effects of Pompia essential oil, coupled with its anti-senescence properties, suggest its potential as a therapeutic agent for enhancing wound repair, thus opening new avenues for the treatment of chronic wounds and age-related skin conditions. Nevertheless, further studies are needed to validate these findings, as cell lines do not fully replicate the complexity of the skin’s three-dimensional structure, nor do they account for the interactions of other cell types, such as keratinocytes, or the influence of the immune system.

## 4. Materials and Methods

### 4.1. Plant Material and Preparation

Pompia fruits were collected during the 2023–2024 harvest season in *Citrus* groves of Irgoli (NU, Italy), in the north-east of Sardinia Island. To ensure a representative sample, over 30 kg of plant material was harvested, corresponding to 84 fruits. Botanical identification was performed at the laboratory of Economic and Pharmaceutical Botany at the University of Cagliari.

Fruits designated for essential oil extraction were immediately processed upon collection. The flavedo was carefully separated from the underlying albedo and pulp and subjected to hydro-distillation [53]. The fruits intended for histological investigation were stored under controlled conditions (cool, dark environment) for 24 h prior to fixation and subsequent microscopic examination.

### 4.2. Histological Analysis

Three fruits were utilized for the histological visualization of the secretory glands. For each fruit, one sample was taken from the upper part of the fruit, adjacent to the petiole, one from the lower part, and one from the side. The internal pulp was removed, and the external part, constituted by flavedo and albedo, was fixed in 70% (*v*/*v*) ethanol. Samples were then dehydrated through an ethanol series and embedded in Technovit 7100 resin (Heraeus Kulzer, Wehrheim, Germany). Longitudinal and cross sections, each 12 µm thick, were obtained with a HM 355 S microtome (Epredia™, Kalamazoo, MI, USA), stained with 0.05% toluidine blue and examined under a Leica DMRB light microscope (Leica Microsystems, Wetzlar, Germany). Pompia sections were compared with analogous histological sections of *Citrus maxima*, also characterized by a high internal albedo and well-documented secretory cavities [54]. Sample size and the number of replicates for *C. maxima* were aligned to those used for Pompia samples to ensure consistency in the comparative analysis.

### 4.3. GC-MS Analysis

The obtained essential oil was then analyzed by gas chromatography (GC) and gas chromatography/mass spectrometry (GC/MS). GC analyses were performed using a gas chromatograph (Agilent 7890A, Palo Alto, CA, USA), equipped with a 30 m × 0.25 mm i.d. HP-5 capillary column (Agilent J&W, Santa Clara, CA, USA) with 0.25 µm stationary film thickness. The analysis followed the operational program previously reported [55]. Briefly, 1 μL of the diluted sample (1:100 *w*/*w* in n-hexane) was injected using an autosampler (Agilent, Model 7683B, Santa Clara, CA, USA), with a 1:20 split ratio. The gradient temperature started at 60 °C, increasing at 3 °C min^−1^ to 246 °C over 62 min, followed by a 20 min hold at 246 °C, resulting in a total run time of 82 min. Helium (purity ≥ 99.9999%—Air Liquide, Milan, Italy) was used as the carrier gas at a constant flow rate of 1.0 mL min^−1^. The injector and detector temperatures were maintained at 250 °C and 300 °C, respectively.

GC-MS analyses were performed using an Agilent 6890N gas chromatograph (Santa Clara, CA, USA) equipped with a 30 m × 0.25 mm i.d. HP-5 capillary column (0.25 µm film thickness; Agilent J&W, Santa Clara, CA, USA) coupled to an Agilent 5973 mass selective detector equipped with an electron ionization (EI) source and a quadrupole analyzer (Santa Clara, CA, USA). The temperature program and chromatographic conditions, excluding the detector settings, were identical to those employed for GC-FID analysis. MS parameters were as follows: transfer line temperature 240 °C; ion source temperature of 200 °C with 70 eV ionization energy; quadrupole temperature of 150 °C; scan rate of 3.2 scans s^−1^ over m/z scan range of 30–480. Chromatograms and mass spectral data were processed using MSD ChemStation software (Agilent, rev. E.01.00.237, Santa Clara, CA, USA). Compound identification was achieved by comparing mass spectra with the NIST and Adams libraries [23,24] and further validated by comparing compounds’ elution order with their retention indices on semi-polar phases [23,24]. Retention indices were calculated relative to the retention times of a series of *n*-alkanes (C8–C20 and C21–C40 standards) using linear interpolation [56]. Relative percentage of individual components was determined from GC peak areas without applying FID response factor correction. Results are presented as percentage of individual peaks ± standard deviation of two independent chromatographic runs.

### 4.4. Cell Culture

The NIH/3T3 cell line (mouse embryonic fibroblast, ATCC CRL-1658), a widely used model in tissue repair studies, due to its typical fibroblast features, was obtained from the American Type Culture Collection. Cells were cultured in Dulbecco’s Modified Eagle’s Medium (DMEM) supplemented with 10% fetal bovine serum (FBS) and 1% penicillin/streptomycin and incubated at 37 °C in a humidified incubator with 5% CO_2_. Experiments were conducted when cultures reached approximately 75–80% confluence, and fibroblast morphology was confirmed via optical microscopy.

### 4.5. Cell Viability

The effect of different concentrations of Pompia essential oil on fibroblast viability was evaluated through the resazurin reduction test, as previously reported [57]. Briefly, NIH/3T3 fibroblasts (50,000 cells/mL) were seeded in 48-well plates. Following overnight stabilization, cells were exposed to essential oil concentrations ranging from 800 to 3.125 µg/mL for 24 h. At the end of the incubation, the culture medium was replaced with fresh medium containing 5 µM resazurin, and cells were further incubated for 2 h. Absorbance was measured at 570 nm, with a reference filter at 620 nm registered in an automated plate reader (SLT, Austria). Cell viability was calculated using the following equation:

Cell viability (%) = (Abs_Exp_/Abs_CT_) × 100;

where Abs_Exp_ is the absorbance difference (570–620 nm) under experimental conditions and Abs_CT_ corresponds to the absorbance of untreated control cells.

### 4.6. Scratch Wound Assay

Cell migration was assessed using the scratch wound assay [58] with slight modifications, as previously reported [59]. Briefly, NIH/3T3 fibroblasts were seeded at 300,000 cells/mL and allowed to reach confluency. Afterwards, the wound was inflicted with a 200 µL pipette tip, and non-adherent cells were removed by washing with PBS pH 7.4. DMEM with 2% FBS with or without the essential oil (12.5–3.125 μg/mL) was added to the cells. Images were acquired at 0 and 18 h post-scratch using a phase-contrast microscope, and the wound area was measured using an ImageJ/FIJI plugin [60]. The results presented were obtained using the following equation:

Wound closure (%) = (A_t=0h_ − A_t=18h_)/A_t=0h_ × 100;

where A_t=0h_ is the area of the wound 0 h after the scratch, and A_t=18h_ is the area 18 h post-scratch.

### 4.7. Cellular Senescence

Cellular senescence was induced by etoposide. Briefly, NIH/3T3 cells were plated at the mentioned concentrations and left to adhere overnight. Then, etoposide (12.5 µM) was added for 24 h. After this time, the medium was removed, fresh medium was added and cells were left to recover for 7 days, in the presence or absence of Pompia EO (12.5 µg/mL).

### 4.8. Senescence-Associated β-Galactosidase Activity

NIH/3T3 cells were plated at 1500 and 20,000 cells/mL for control and etoposide-treated cells, respectively. SA-β-galactosidase activity was determined using a commercially available kit according to the manufacturer’s protocol (#9860, Cell Signaling Technology, Inc., Danvers, MA, USA). Cells were imaged for quantitative analysis using ImageJ software, with senescent cells presenting the characteristic blue stain, indicative of β-galactosidase activity. Results were presented as percentages of SA-β-gal-positive cells. A minimum of 100 cells were acquired in each independent experiment.

### 4.9. γ.H2AX Immunofluorescence Staining

NIH/3T3 cells were cultured on glass coverslips at 1500 and 20,000 cells/mL for control and etoposide-treated cells, respectively, and treated as reported in Section 4.8. Cells were then fixed with 4% paraformaldehyde (PFA) for 10 min and washed with PBS. Cells were permeabilized with 0.1% Triton X-100 for 15 min followed by washes with PBS and a blocking step with blocking solution (3% bovine serum albumin and 10% goat serum in PBS) for 1 h. γH2AX puncta were detected using a primary antibody (1:500, #9718 Cell Signaling) applied to cells overnight at 4 °C, in blocking solution. Afterwards, cells were washed three times with PBS and incubated for 1 h at room temperature with a secondary antibody (1:500, goat anti-rabbit Alexa Fluor 568) and DAPI (1:1000), made in blocking solution. Following washes with PBS, coverslips were mounted on glass slides with Mowiol mounting medium. Images were obtained using a confocal point-scanning microscope (Zeiss LSM710; Carl Zeiss, Oberkochen, Germany) with a 63× objective. The number of puncta per nucleus was determined using the Yen threshold setting with manual adjustment of ImageJ v1.54p and counted automatically using the “analyze particles” function of ImageJ.

### 4.10. Statistical Analysis

Statistical analyses were conducted using R software (version 2023.06.1+524) [61] with agricolae, car, and ggplot2 packages. Prior to analysis, data were assessed for normality and homogeneity of variances, using the Shapiro–Wilk test and Levene’s or Bartlett’s test, respectively. Depending on the outcome, either parametric or non-parametric tests were applied to evaluate the histological data (ANOVA fb post hoc Tukey’s test for parametric data; Kruskal–Wallis fb Wilcoxon test for non-parametric data). Regarding bioactive assays, one-way analysis of variance (ANOVA) followed by the appropriate post hoc test was performed using GraphPad Prism version 9.3.0; *p*-values < 0.05 were accepted as statistically significant. For a comprehensive understanding of the main findings, the *p*-value, alongside the R-squared value and confidence intervals of the mean differences, is provided.

## 5. Conclusions

This study provides the first integrated histological, chemical and biological characterization of the Pompia fruit, an old endemic and culturally significant *Citrus* ecotype from Sardinia. The histological analysis revealed distinctive features in the peel, such as smaller and more deeply embedded secretory cavities and epidermal irregularities, which may be linked to its irregular morphology and low pulp content. Chemical analysis confirmed the essential oil’s richness in bioactive monoterpenes, particularly limonene. The biological assays demonstrated promising wound healing and anti-senescence activities in vitro, suggesting potential applications in the cosmetic sector, for the treatment of chronic wounds or age-related skin conditions. Overall, these findings offer new insights into the anatomical and functional properties of Pompia, laying the groundwork for future studies and the valorization of this traditional fruit in both the agronomic and therapeutic fields.

## Figures and Tables

**Table 1:** Compounds of Pompia essential oil identified by GC-MS.

RI	RI (Litt)	Compound	% Area ± SD
933	932	α-Pinene	0.45 ± 0.008
973	969	Sabinene	0.05 ± 0.002
977	974	β-Pinene	0.05 ± 0.001
986	986	6-Methyl-5-hepten-2-one	0.04 ± 0.002
991	988	Myrcene	1.71 ± 0.047
1003	998	*n*-Octanal	0.03 ± 0.002
1006	1002	α-Phellandrene	0.08 ± 0.002
1026	1024	Limonene	89.17 ± 0.939
1036	1038	*cis*-Ocimene	0.17 ± 0.031
1047	1047	*trans*-Ocimene	0.76 ± 0.023
1058	1054	γ-Terpinene	0.04 ± 0.001
1069	1063	*n*-Octanol	0.04 ± 0.005
1089	1086	Terpinolene	0.05 ± 0.001
1100	1095	Linalool	0.47 ± 0.029
1104	1100	*n*-Nonanal	0.56 ± 0.033
1153	1148	Citronellal	0.22 ± 0.018
1183	1177	(*E*)-Isocitral	0.05 ± 0.004
1191	1186	α-Terpineol	0.16 ± 0.008
1228	1223	Citronellol	0.82 ± 0.109
1241	1235	Neral	1.24 ± 0.093
1254	1249	Geraniol	0.35 ± 0.057
1271	1264	Geranial	1.63 ± 0.170
1274	1269	Perilla aldehyde	0.06 ± 0.001
1365	1359	Neryl acetate	0.17 ± 0.023
1385	1379	Geranyl acetate	0.12 ± 0.020
1420	1420	β-Caryophyllene	0.10 ± 0.013
1436	1432	α-*trans*-Bergamotene	0.43 ± 0.046
1457	1454	*E*-β-Farnesene	0.03 ± 0.004
1493	1496	Valencene	0.27 ± 0.031
1497	1500	Bicyclogermacrene	0.08 ± 0.010
1509	1505	β-Bisabolene	0.59 ± 0.064
**Total identified**			**99.97**
**Total monoterpenes**			**92.53**
**Total sesquiterpenes**			**1.50**
**Total oxygenated**			**5.4**

In the table, RI refers to the retention index experimentally determined on an HP-5 ms fused silica column relative to a series of n-alkanes; RI (Litt) refers to the retention index reported in the literature [23,24]. Reported compounds were identified by comparing their mass spectra (MS) and retention indices (RI) with those reported in Adams and NIST libraries [23,24].

## Data Availability

The original contributions presented in this study are included in this article and the Supplementary Materials. Further inquiries can be directed to the corresponding author.

## Supplementary Materials

The following supporting information can be downloaded at https://www.mdpi.com/article/10.3390/ph18091256/s1: Figure S1: Pompia EO chromatogram.
