# Supplementary material for: Histology of Pompia Peel and Bioactivity of Its Essential Oil: A New Citrus-Based Approach to Skin Regeneration
Source: Pharmaceuticals (Basel). 2025 Aug 24;18(9):1256. doi: 10.3390/ph18091256 (PMC12472668; doi:10.3390/ph18091256)
Supplement: Supplementary file 1 [file pharmaceuticals-18-01256-s001.zip › Figure S1.pdf]

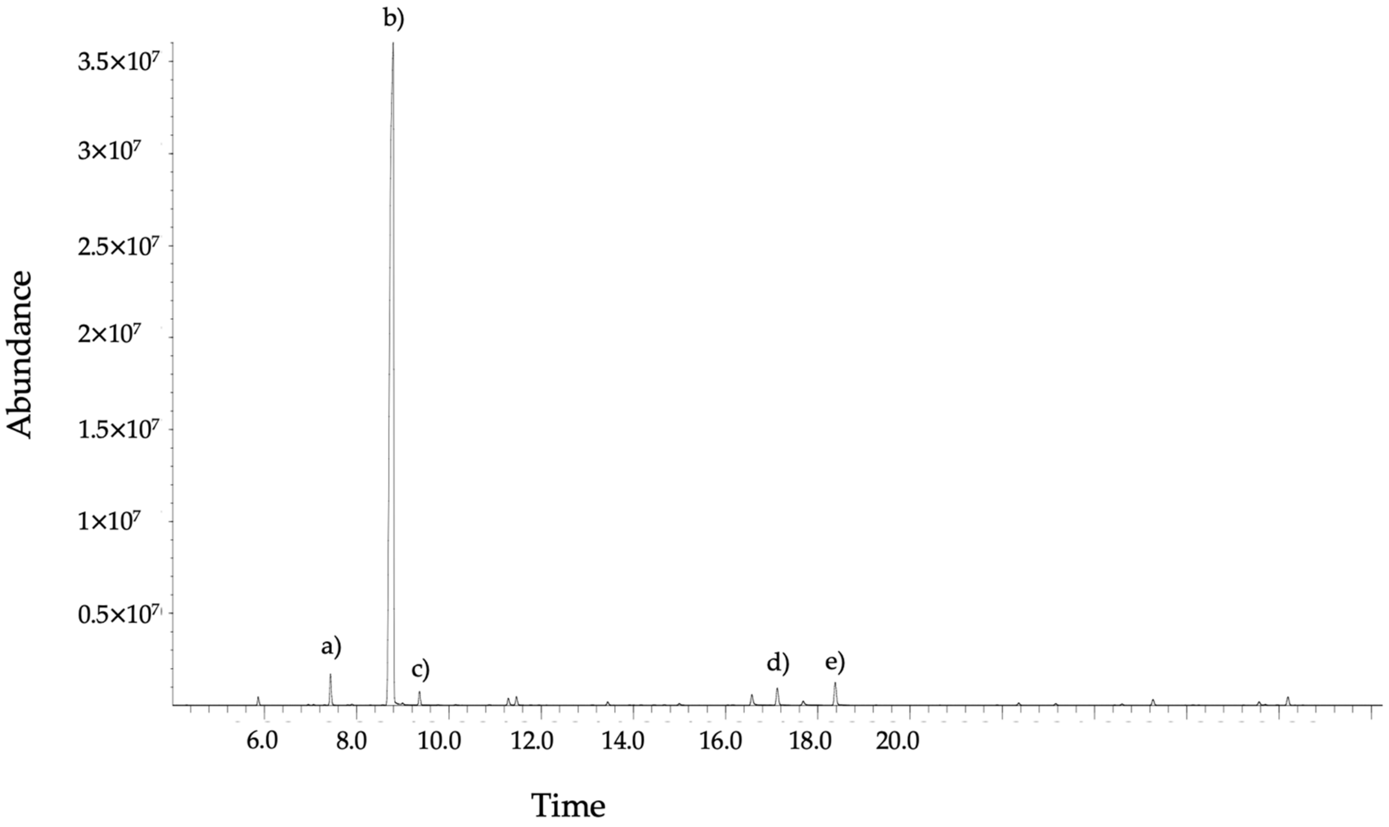

Figure S1. GC–MS chromatogram of Pompia essential oil. The X-axis represents retention time (minutes). Letters indicate the most abundant compounds: (a) myrcene; (b) limonene; (c) trans-ocimene; (d) neral; (e) geranial. All identified compounds and their relative peak area percentages are reported in Table 1.

.
